# Development of Ovarian Tissue Autograft to Restore Ovarian Function: Protocol for a French Multicenter Cohort Study

**DOI:** 10.2196/12944

**Published:** 2019-09-30

**Authors:** Jean-Baptiste Pretalli, Sophie Frontczak Franck, Lionel Pazart, Christophe Roux, Clotilde Amiot, DATOR Group

**Affiliations:** 1 INSERM CIC 1431 Centre d'Investigation Clinique Centre Hospitalier Universitaire de Besançon Besançon France; 2 Department of Reproductive Medicine and Biology, Cryobiology University Hospital of Besançon Besançon France; 3 EA481 - Integrative and Clinical Neuroscience Laboratory University Bourgogne Franche-Comté Besançon France; 4 INSERM, Établissement Français du Sang Bourgogne Franche-Comté, UMR1098 Interactions Hôte-Greffon-Tumeur/Ingénierie Cellulaire et Génique University Bourgogne Franche-Comté Besançon France

**Keywords:** cohort study, ovarian tissue, cryopreservation, fertility preservation, pregnancy rate, live birth rate

## Abstract

**Background:**

Sterility is a major late effect of radiotherapy and chemotherapy treatments. Iatrogenic sterility is often permanent and greatly impacts long-term quality of life. Ovarian tissue cryopreservation (OTC) performed before gonadotoxic treatments with subsequent autograft is a method of fertility preservation available for girls and women. Its application in prepubertal girls is of particular value as it is the only possible approach in this patient group. In addition, it does not require a delay in cancer therapy and no ovarian stimulation is needed.

**Objective:**

The primary aim of this protocol is to help increase the implementation of ovarian tissue autografting in France. Knowledge is still lacking regarding the efficacy of ovarian transplantation in restoring ovarian function and regarding the safety of this procedure, especially the risk of cancer cell reseeding in certain types of cancer. A secondary aim of this study is to generate data to improve our understanding of these two essential aspects.

**Methods:**

The DATOR (Development of Ovarian Tissue Autograft in Order to Restore Ovarian Function) study is ongoing in 17 university hospitals. The DATOR protocol includes the autograft of ovarian cortex fragments. Candidates are identified from an observational prospective cohort (called the Prospective Cohort of Patients Candidates for Ovarian Tissue Autograft [PERIDATOR]) of patients who have undergone OTC. Enrollment in the study is initiated at the patient’s request and must be validated by the center’s multidisciplinary team and by the study steering committee. The DATOR study begins with a total medical checkup. Ovarian tissue qualification and residual disease detection, if required, are performed.

**Results:**

The study is ongoing. Currently, 38 patients have provided informed consent and have been entered into the DATOR study. Graft has been performed for 34 of these patients. An interim analysis was conducted on the first 25 patients for whom the period of at least 1 year posttransplantation was achieved. Out of these 25 patients, 11 women succeeded in becoming pregnant (pregnancy rate=44% [11/25]; delivery rate=40% [10/25]). Among these, 6 women conceived twice, and 1 pregnancy led to a miscarriage.

**Conclusions:**

Our preliminary analysis appears to be coherent with the accumulating body of evidence indicating the potential utility of ovarian tissue autograft for patients with premature ovarian failure. All these elements justify the pursuit of our study.

**Trial Registration:**

ClinicalTrials.gov NCT02846064; https://clinicaltrials.gov/ct2/show/NCT02846064

**International Registered Report Identifier (IRRID):**

DERR1-10.2196/12944

## Introduction

Cancer is a major public health issue. Its incidence is increasing worldwide, particularly in adolescents and young adults [1-3]. Chemotherapy and/or radiotherapy have significantly improved the chances of long-term survival [4-9]. For women of reproductive age, cancer treatments, particularly those using alkylating agents, and radiation therapy directed at the pelvis or the abdomen can incur the major side effect of inducing premature ovarian failure and infertility [10-16]. The incidence of iatrogenic sterility is consequently increasing [17]. The risk is linked to the patient’s age, as well as the type, amount and timing of the treatment delivered [13,18,19].

Mechanisms by which chemotherapies induce damages to ovarian reserve are partially elucidated. Treatments could deplete the primordial follicle pool by direct toxicity to follicles and/or by increasing primordial follicle activation into growing follicles [20]. In cases of pelvic irradiation, preventive surgical measure such as oophoropexy may be indicated. Nonsurgical measures (implying gonadotropin-releasing hormone or luteinizing hormone (LH)–releasing hormone inhibition) designed to minimize gonadotoxic effects might represent alternative strategies, whose efficiency is still controversial [21-28]. Recent studies indicate that primordial follicle activation modulators may provide another promising option for fertility preservation in cancer patients for whom oocyte and embryo cryopreservation are not possible. Nevertheless, they are still under trial [29-31]. A decrease in or loss of fertility is a traumatic issue that greatly impacts long-term quality of life. Several studies have reported the emotional distress of cancer survivors that became sterile [32-34]. Although a spontaneous return of ovarian function and fertility is possible, it occurs in only very few patients [35,36]. In this context, most health care providers recognize the importance of fertility preservation measures before the initiation of anticancer drugs. Nevertheless, a very small proportion of patients actually benefit from available fertility preservation procedures [37-44].

Several options can be proposed according to the patients’ age, marital status, and pathology. One such fertility preservation technique is ovarian tissue cryopreservation (OTC) [45,46]. OTC is independent of ovarian stimulation and can therefore be implemented without delay. In addition, it is the only technique that can be offered to children because neither a partner nor ovarian stimulation with a pickup are required, and it is also suitable for women with hormone-sensitive cancer [12,47,48]. Laparoscopic harvesting consists of a total (unilateral) or partial oophorectomy or ovarian tissue biopsies. Ovarian cortex is frozen in fragments (1 cm/0.5 cm) according to a protocol using slow cooling with manual or automatic seeding [49,50] and then stored in nitrogen gas or liquid. Rapid freezing (vitrification) of ovarian tissue is also possible and might even be more effective than slow freezing [51-53]. Fertile oocytes can be obtained from fragments of cryopreserved ovarian cortex only by maturation of the oocytes present in the primordial, primary, and preantral follicles that have withstood the freezing-thawing process. Various research teams are working on developing in vitro folliculogenesis techniques [54-62] but also autologous transplantation of isolated follicles and subsequent ovarian reconstruction [63-67].

Ovarian tissue autograft is currently the only technique allowing natural restoration of fertility and ovarian endocrine function [68]. Once full cancer recovery is achieved, thawed tissues can be transplanted back into the patient who wishes to have a baby [69]. Ovarian tissue can be transplanted orthotopically (ie, into the pelvic cavity in or near the remaining ovary) or heterotopically (ie, extrapelvically into the forearm or the abdomen for example), the former technique being associated with greater success rates [70-73].

The first ovarian transplantations were reported by Oktay and Karlikaya in 2000 and Radford et al in 2001 [74,75]. These researchers showed a resumption of follicular development, with or without ovarian stimulation. The first human embryo was obtained in vitro in 2004 after heterotopic grafting of subcutaneous ovarian tissue, but its development stopped after its transfer into the uterine cavity [76]. The first live birth after orthotopic autotransplantation of cryopreserved ovarian tissue was described in 2004 by Donnez et al [77]. Ovarian tissue transplantation has since resulted in the birth of more than 130 babies [78,79]. Delivery rates are usually used as an indicator for success, even if not perfectly accurate (there may be more than one transplantation per woman and not every grafted woman had fertility achievement in mind). Delivery rates reported are from 15% to 50% [68,79-88].

Predictive factors that would allow the identification of the patients most likely to conceive and safely deliver healthy babies after autotransplantation have not yet been identified. Therefore, there is a need for cohort studies following patients from OTC through to the babies’ first months.

In 2009, our team, in collaboration with the university hospital of Limoges (France), reported the first live birth in our country, the seventh in the world. It was the first live birth associated with a noncancerous disorder, namely sickle cell anemia treated by allogeneic bone marrow transplantation [89].

Initially, in France, ovarian tissue preservation was only authorized in the context of research protocols. Then, new legislation on Bioethics (2004) allowed for the practice of germinal tissue preservation in centers accredited by the BioMedicine Agency (Agence de BioMedecine, ABM; decree of December 22, 2006). Worldwide, thousands of patients have benefited from OTC. In France, the ABM reported that 2845 patients had their ovarian tissue cryopreserved by the end of 2016. In France, the 2008 decree relative to the rules for good clinical and biological practices for medically assisted procreation stipulates that the use of germinal tissue must remain in the field of research. A further decree published in 2017 requires that specific information on the knowledge and results generated by any research protocols must be delivered [90].

Only 3 research protocols are currently recruiting patients in our country with the aim of proposing transplantation of cryopreserved ovarian cortex in women with premature ovarian failure. One of those protocols, named CAROLéLISA (*Autograft of Human Ovarian Tissue: Efficiency and Safety*), is led by Professor Catherine Poirot in the unit of reproduction biology of Assistance Publique–Hôpitaux de Paris in Paris. The first patient was included in June 2010 and the cohort currently comprises 40 subjects. Professor Bruno Salle and Dr Jacqueline Lornage are leading a protocol in the university hospital Lyon-Bron, Lyon, but no details of the study procedures are available.

The DATOR study (Development of Ovarian Tissue Autograft in Order to Restore Ovarian Function) (NCT02846064) was launched in 2013 with the aims of assessing the safety and efficacy of ovarian tissue autotransplantation in terms of restoration of ovarian function and fertility. This evaluation will take into account graft survival in the short-to-medium term.

## Methods

### Design and Objectives

The DATOR study is a multicenter prospective longitudinal cohort project. Candidates for the DATOR study are identified from an observational prospective cohort (named Prospective Cohort of Patients Candidates for Ovarian Tissue Autograft [PERIDATOR]) of patients who have undergone OTC. The PERIDATOR cohort specifically aims to record the number and describe the profile of patients who have undergone OTC in the participating hospitals. The DATOR study allows the dissemination of the orthotopic 2-step surgical technique in France, thus making it available to a greater number of patients.

### Participants

The DATOR protocol was initially conducted in 12 French university hospitals, and a total of 17 centers are now recruiting (Besançon, Limoges, Toulouse, Rouen, Bordeaux, Clermont-Ferrand, Strasbourg, Clamart, Nantes, Marseille, Lille, Reims, Bondy, Grenoble, Nancy, Tenon, and Poissy). In each center, each case is discussed within a multidisciplinary team including biologists, surgeons, gynecologists, hematologists, oncologists, pathologists, endocrinologists, and/or pediatricians.

### Study Size

After a preliminary survey of the 12 centers that were among the first to participate, the number of patients eligible was estimated at 186, out of a total of 650 cryopreservations performed in those centers at that time. Recruitment began in 2013.

Regarding the DATOR study, an authorization was originally obtained for 10 transplantations over a 2-year period. A first protocol modification extended the number of grafts allowed to 20. Two 24-month extensions of the inclusion period were granted in 2017 and in 2018. Therefore, the inclusion period now extends from 2013 to 2021 with a 3-year follow-up to achieve 62 ovarian transplantations.

### Recruitment

The inclusion criteria for both the PERIDATOR cohort and the DATOR study are as follows: women between 18 and 43 years of age, who underwent ovarian tissue cryopreservation, and cured of their primary disease.

Specific inclusion criteria for the PERIDATOR cohort are as follows: short- or medium-term childbearing desire and premature ovarian failure documented by ultrasound criteria (antral follicle counts <5) and hormonal criteria (anti-Müllerian hormone [AMH] <2 ng/mL, even if follicle-stimulating hormone [FSH] <20 IU/L).

Specific inclusion criteria for the DATOR study are as follows: short-term childbearing desire; total premature ovarian failure defined, in the absence of hormone therapy, by the association of suggestive clinical criteria (secondary amenorrhea, flushes, signs of estrogen deficiency, etc), ultrasound criteria (absence of antral follicle), and hormonal criteria: FSH >20 IU/L, AMH <2 ng/mL, and low estradiol level; and patients included retrospectively, that is, patients who have already undergone ovarian tissue autograft.

Exclusion criterion is patients under legal protection.

### Inclusion in the Development of Ovarian Tissue Autograft in Order to Restore Ovarian Function Study

Patient included in the PERIDATOR cohort are followed up annually after OTC and after the end of the gonadotoxic therapy (clinical, hormonal, and ultrasound workup with the biologists and gynecologists in the treating center). Adult patients who wish to conceive a child can make a request to undergo ovarian tissue autograft. Participation in the DATOR study is then proposed, and if the patient accepts, then the case is discussed in the multidisciplinary meeting in the treating center.

#### Pretransplantation Workup and Biological Qualification of the Grafts

The clinical examination includes the search for clinical signs of primary ovarian insufficiency (absence of menstrual cycles and hot flushes). The administration (or not) of hormone replacement therapy is recorded. The mandatory blood tests required for medically assisted procreation as well as hormone tests (FSH, LH, AMH, inhibin B, and estradiol) are performed. Pelvic ultrasound with Doppler is performed to assess the size of the uterus and the remaining ovary (-ies) and the thickness of the endometrium under hormone replacement therapy. Finally, a preoperative consultation with an anesthesiologist is also performed.

To complete the pretransplant workup, biological qualification of the grafts is performed. One of the fragments of cryopreserved ovarian tissue, selected at random, is thawed according to the protocol validated for the actual transplant. The majority of the thawed fragment is then prepared for pathological examination, and the remainder is used to evaluate the long-term viability of the isolated ovarian follicles. The pathology exam evaluates the quality of the tissue after the freeze-thaw cycle and the abundance of follicles, and it also investigates the presence of any potential anomalies, in particular the presence of residual malignant cells in cases where the initial pathology was cancer. If the number and quality of follicles are found to be low, then the number of fragments of ovarian cortex to be used for the autograft can be increased. Microbiological controls are performed on the freezing, thawing, and transport media of the fragment under investigation.

The possible reseeding of malignant cells during the autograft of ovarian fragments is a problem of great importance. Acute leukemia is the most common childhood cancer. Performing autotransplantation on patients at high risk of cancer reseeding (ie, acute leukemia) is not recommended because of the high risk of cancer cell reintroduction [91,92]. The clinical decision requires the use of minimal residual disease detection techniques, molecular analysis [93-95], flow cytometry [96,97], and xenograft [98,99].

#### Decision of the Steering Committee

The local investigating centers put together a file that is subsequently submitted to the steering committee of the DATOR study. The steering committee is composed of experts from the multidisciplinary teams of various university hospitals in France. The steering committee evaluates the rational for the autograft, and the risk-benefit ratio (quality of the preserved fragments, obstetrical risk, and cancer risk). The steering committee may call on outside experts, if necessary. The decision of the steering committee takes account of the progress in techniques that make it possible to assess residual disease in the ovaries. After evaluation of the file, the steering committee approves (or does not approve) the ovarian transplantation.

### Development of Ovarian Tissue Autograft in Order to Restore Ovarian Function Protocol

#### Graft Thawing and Preparation

The freezing and thawing protocol for ovarian tissue has previously been described and established in the laboratory [89]. The thawing of the ovarian fragments is carried out sterilely in class A, under a hood, in the laboratory of the assisted reproductive technology (ART) center. After quickly thawing the vials, the strips are washed in decreasing solutions of DMSO 1.5 M (5 min), 1 M (5 min), 0.5 M (10 min), and 0.05 mol/L sucrose in Leibovitz L-15 medium supplemented with 10% decomplemented patient serum. The strips are then rinsed and transferred to the operating theater for the graft in medium containing 20% serum only.

Regarding the second step of the transplantation, if the size of the fragments of ovarian cortex is less than 0.5 cm², several fragments can be sutured in an *ovarian patch* of 2 cm² by the surgeon in the laboratory under a laminar flow hood.

#### Autograft

Surgeons from each of the centers participating in the study have been trained in the autograft technique for the implantation of cryopreserved ovarian tissue by Dr Pascal Piver who proctored the first autografts in all centers.

The 2-stage orthotopic graft is the first-line technique used in this study [89]. The transplantation is performed in 2 stages by celioscopy under general anesthesia. It can also be performed with the aid of a surgical robot in centers equipped with such facilities and who are trained in its use. Heterotopic transplantation can be proposed if a contraindication exists to orthotopic transplantation.

#### Follow-Up in the Development of Ovarian Tissue Autograft in Order to Restore Ovarian Function Study

To collect outcome data to evaluate the success of the autograft, monthly follow-up is performed for 1 year after the transplant or until the patient becomes pregnant. The monthly follow-up includes clinical, biological, and ultrasound examination. The clinical evaluation is performed by the gynecologists and biologists of the ART unit in the participating center. They investigate for clinical signs of recovery of ovarian function (onset of spontaneous menstrual cycles or disappearance of hot flushes). Biological follow-up includes hormone tests (FSH, LH, AMH, inhibin B, estradiol, and progesterone). Normalization of endogenous gonadotropins is also investigated. While awaiting this stage, hormone replacement therapy and administration of vitamin are pursued.

Echographic follow-up comprises transvaginal ultrasound of the grafts (presence of antral follicles) associated with Doppler examination to evaluate neovascularization. Magnetic resonance imaging is performed 3 months after the transplantation to assess the state of revascularization of the grafts and to determine the origin of neovascularization.

As soon as ovary function is recovered, either by normalization of gonadotropins, with or without elevation of AMH, or by the appearance of echographic signs, monitoring of folliculogenesis and ovulation is implemented at each cycle, with a view to achieving pregnancy by scheduled intercourse, with or without ovulation-stimulating treatment. In case of failure, an appropriate ART technique is implemented (intrauterine insemination or in vitro fertilization with or without intracytoplasmic sperm injection).

Monthly follow-up can be discontinued 1 year after the transplantation if the ovarian function has not recovered. In this case, the transplant is considered to have failed. In this situation, a further transplant attempt with the remaining cryopreserved fragments can be proposed with the approval of the steering committee.

Finally, if the patient becomes pregnant, follow-up is completed by a search for any complications during pregnancy and delivery, and the baby’s development is followed up to 3 months after the birth.

**Figure 1:**
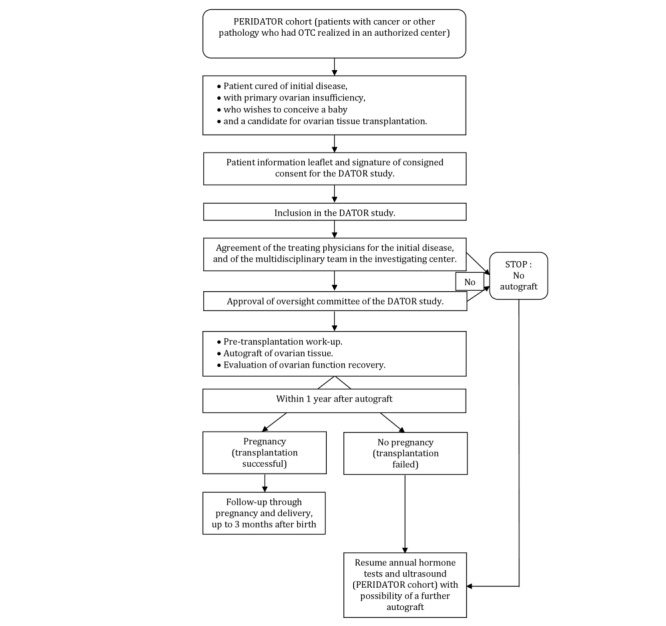
Study flowchart.

The study flowchart is presented in Figure 1.

### Data Analyzed

#### Primary Outcome

Patients will be dichotomized into 2 groups. The *restored* group will comprise patients who achieve ovarian function restoration evidenced by the onset of a pregnancy. The *not restored* group will comprise patients who do not achieve ovarian function restoration. Fertility restoration is defined as the occurrence of a pregnancy leading or not leading to a live birth.

#### Secondary Outcomes

The secondary outcomes are as follows: number of live births after ovarian tissue autograft, number of complications that could result from a surgery with anesthesia or depending on graft quality, number of graft recovery, and number of residual disease development.

The information obtained with this study will contribute to our knowledge on autologous ovarian tissue transplantation, the preservation of ovarian function, and the reuse of self-preserved ovarian tissue to be improved. Advances are also expected in the management of patients.

This protocol will allow the surgical teams of the participating centers to be trained in the practice of the autograft of ovarian cortex, especially the 2-stage grafting technique codified by Limoges’ team.

### Data Collection and Research Measures

Data pertaining to all patients will be rendered anonymous before being centralized at the coordinating center in Besancon, where they will be verified and completed (if necessary) and entered into a secure database. All study documentation will be conserved in a locked office.

### Statistical Methods

An interim analysis was planned on the first 25 patients who arrived at 1 year after transplantation. The objective was to check that the results were sufficiently favorable to allow continuation and extension of the study.

All analyses will be performed using SAS version 9.4 (SAS Institute Inc). Continuous variables will be presented as mean (SD) and median (interquartile range). Categorical variables will be presented as number and percentage.

Some potential prognostic factors will be measured at baseline and will be compared between the *not restored* fertility and *restored* fertility groups using the Pearson chi-square test or Fisher exact test for categorical variables and the Student *t* test or analysis of variance for normally distributed quantitative variables. The Mann-Whitney U and Kruskal-Wallis tests will be used for comparison of nonnormally distributed variables and semiquantitative variables. Multivariate analysis (logistic or Cox regression) will be used if we have a sufficient power for this analysis. A *P* value of less than .05 will be considered statistically significant.

### Ethics and Dissemination

This study is conducted in agreement with the Declaration of Helsinki (amended in October 2013). Before study initiation, the protocol and informed consent document were submitted to the ethical review committee of Franche-Comté and to the French National Agency for the Safety of Health Products (*Agence nationale de sécurité des médicaments et des produits de santé*), both of which gave their approval. Signed informed consent is obtained from all participating patients. Patient anonymity is protected by the use of subject identification codes.

Clinical follow-up is provided by physicians and surgeons. The risks associated with ovarian tissue transplantation are the risks related to laparoscopy and general anesthesia. No drugs other than those normally prescribed in daily medical practice are used.

The findings from this study will be disseminated at several regional and international research conferences and as published articles in peer-reviewed journals.

The trial is registered with Clinicaltrials.gov under the number NCT02846064.

## Results

### Project Progress

**Figure 2:**
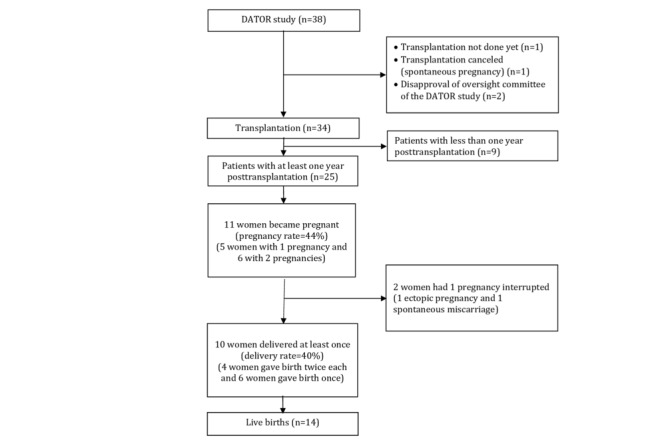
Flowchart of the preliminary analysis. DATOR: Development of Ovarian Tissue Autograft in Order to Restore Ovarian Function.

Recruitment is ongoing. A total of 142 patients have been included in the PERIDATOR cohort (data as of December 31, 2018), and 38 patients are now cured of the initial disease and have provided written consent to enter the DATOR study. Transplantation has been performed in 34 of them. The flowchart of the preliminary analysis is presented in Figure 2.

### Preliminary Analysis

An interim analysis was planned on the first 25 patients for whom the period of at least 1 year post transplantation was achieved. Among these 25 patients, 11 women succeeded in becoming pregnant (pregnancy rate=44%). In addition, 10 women gave birth to 14 babies, 4 of them conceived twice (delivery rate=40%), and two-third (11/17) of them resulted from natural conception. The average time to conception was 11.3 (SD 6.3) months, with a minimum of 5.7 months and a maximum of 25.5 months.

Patients’ characteristics and outcomes of transplantation are presented in Tables 1 and 2. No heterotopic transplantation was performed.

Menses returned spontaneously in 68% of the patients (n=14), on average 5.4 months (SD 2.3) after ovarian tissue autograft (minimum=2, maximum=93; n=22). Antral follicles were found in 20 patients of the 21 patients (95%) for whom data were available. The first antral follicle appeared on average 3.7 months (SD 2.2) after ovarian tissue autograft (minimum=1, maximum=10). From the onset of the first antral follicle in ultrasound, the mean count of antral follicles was 3 (SD 1.6; minimum=1, maximum=8).

**Figure 3:**
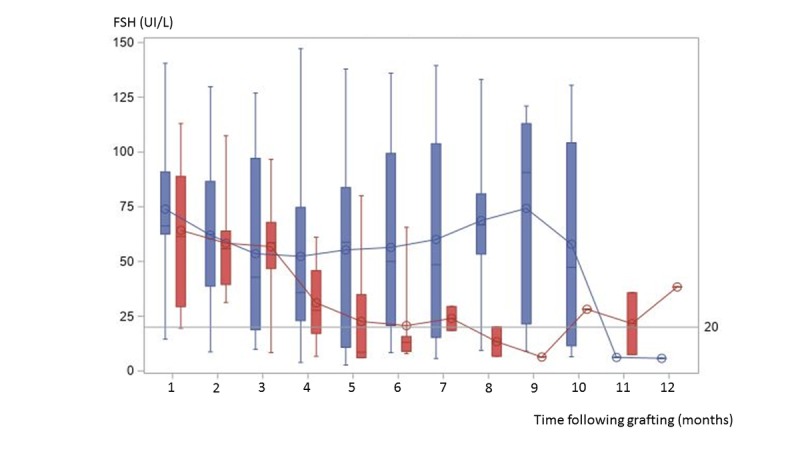
Box plots of monthly follicle-stimulating hormone levels during the year after autograft for the no pregnancy (blue) and the pregnancy group (red). For each group, the curves represent the monthly follicle-stimulating hormone means for the no pregnancy (blue curve) and for the pregnancy group (red curve), respectively. FSH: follicle-stimulating hormone.

The global mean FSH after autotransplantation was 50.1 UI/L (SD 26.7; n=23; minimum=8.2, maximum=133) for all patients, 55 UI/L (SD 32.5; n=14; minimum=8.2, maximum=133) for the *no pregnancy* group and 41 UI/L (SD 9.9; n=9; minimum=32, maximum=65) for the *pregnancy* group, without significant difference. However, a progressive decrease in FSH was observable for the *pregnancy* group at the beginning of the follow-up and more particularly from the third month until falling below the threshold of 20 IU/L on average in the sixth month of follow-up Figure 3.

During the follow-up, AMH was undetectable for 10 patients, detectable for 12, and missing for 3. When positive, on average 3.4 months (SD 0.2) after autograft, the mean level of AMH was 0.3 ng/mL (minimum=0.01, maximum=0.77).

Procedures for the detection and recording of adverse events were implemented. No serious adverse event relating to transplantation and no residual disease reseeding was reported. All babies were born healthy.

**Table 1:** Demographic and clinical characteristics.

Variables		No pregnancy	Pregnancy	Total	*P* value
Patient, n (%)		14 (56)	11 (44)	25 (100)	N/A^a^
**Initial disease, n (%)**					
	Hodgkin lymphoma	11 (79)	6 (55)	17 (68)	N/A
	Non-Hodgkin lymphoma	1 (7)	2 (18)	3 (12)	N/A
	Ewing sarcoma	0 (0)	1 (9)	1 (4)	N/A
	Periarteritis nodosa	0 (0)	1 (9)	1 (4)	N/A
	Systemic mastocytosis	1 (7)	0 (0)	1 (4)	N/A
	Sickle cell disease	0 (0)	1 (9)	1 (4)	N/A
	Neurolupus	1 (7)	0 (0)	1 (4)	N/A
Gonadotoxic treatment before ovarian tissue cryopreservation, n (%)		12 (67)	6 (33)	18 (100)	.99
Age at cryopreservation (years), mean (SD)		26.2 (4.6)	26.7 (3.8)	26.4 (4.2)	.89
Age at transplantation (years), mean (SD)		33.6 (3.9)	31.6 (4.9)	32.8 (4.3)	.81
Storage duration (years), mean (SD)		6.7 (2.5)	5.9 (3.2)	6.4 (2.8)	.29
Number of grafted fragments, mean (SD)		13 (2.6)	11.6 (4.6)	12 (3)	.56
Follicular density/mm^2^, mean (SD)		4.9 (5.2)	5 (5.1)	5 (4.7)	.97
Time to conception (months), mean (SD)		N/A	11.3 (6.3)	N/A	N/A
Follicle-stimulating hormone levels (mUI/mL), mean (SD)		79 (24.8)	86.9 (30.4)	82.5 (27.1)	.43
Anti-Müllerian hormone levels (ng/mL), mean (SD)		0.08 (0.15)	0.03 (0.09)	0.06 (0.13)	.52
Age at conception, mean (SD)		N/A	33.9 (5.2)	N/A	N/A
Live births, n		N/A	14	14	N/A

^a^N/A: not available.

**Table 2:** Detailed patient characteristics and outcomes of transplantation.

Patient number	Initial disease	Treatment before ovarian tissue cryopreservation	Age at grafting (years)	Assisted reproductive technology	Pregnancy	Weeks of amenorrhea	Outcome	Age at conception (years)
1	Polyarteritis nodosa	Yes	35.6	Yes	Yes	N/A^a^	Ectopic pregnancy	36.8
1	Polyarteritis nodosa	Yes	35.6	Yes	Yes	36.6	Live birth	36.1
2	Sickle cell disease	No	22.5	No	Yes	38	Live birth	27.4
2	Sickle cell disease	No	22.5	No	Yes	36	Live birth	29.8
3	Hodgkin lymphoma	Yes	31.6	Yes	Yes	37.9	Live birth	32.2
4	Hodgkin lymphoma	Yes	26.3	No	Yes	40.1	Live birth	27.7
4	Hodgkin lymphoma	Yes	26.3	No	Yes	40.3	Live birth	30.6
5	Hodgkin lymphoma	Yes	37.9	—^b^	No	N/A	N/A	N/A
6	Hodgkin lymphoma	No	33.2	Yes	No	N/A	—	N/A
7	Non-Hodgkin lymphoma	Yes	31	No	Yes	39	Live birth	33.1
8	Ewing sarcoma	No	32.4	No	Yes	41	Live birth	33.5
8	Ewing sarcoma	No	32.4	No	Yes	40.5	Live birth	—
9	Hodgkin lymphoma	Yes	43	Yes	Yes	N/A	Miscarriage	44.5
10	Non-Hodgkin lymphoma	Yes	30.8	Yes	No	N/A	N/A	N/A
11	Hodgkin lymphoma	Yes	27.6	Yes	No	N/A	N/A	N/A
12	Mastocytosis	No	37.2	Yes	No	N/A	N/A	N/A
13	Hodgkin lymphoma	No	38.9	Yes	No	N/A	N/A	N/A
14	Hodgkin lymphoma	No	37.4	Yes	Yes	38.1	Live birth	38
14	Hodgkin lymphoma	No	37.4	No	Yes	—	Live birth	—
15	Hodgkin lymphoma	Yes	31.3	—	No	N/A	N/A	N/A
16	Hodgkin lymphoma	Yes	27.2	Yes	No	N/A	N/A	N/A
17	Hodgkin lymphoma	Yes	28.8	No	Yes	42	Live Birth	29.4
18	Hodgkin lymphoma	No	30.9	No	Yes	41	Live Birth	31.4
19	Hodgkin lymphoma	Yes	34.7	—	No	N/A	N/A	N/A
20	Neurological lupus	Yes	35.4	—	No	N/A	N/A	N/A
21	Hodgkin lymphoma	No	35.6	No	Yes	41	Live birth	36.5
21	Hodgkin lymphoma	No	35.6	No	Yes	N/A	Ongoing pregnancy	N/A
22	Hodgkin lymphoma	Yes	30.1	Yes	No	N/A	N/A	N/A
23	Hodgkin lymphoma	No	34.1	—	No	N/A	N/A	N/A
24	Hodgkin lymphoma	Yes	34.8	—	No	N/A	N/A	N/A
25	Hodgkin lymphoma	Yes	27.3	—	No	N/A	N/A	N/A

^a^N/A: not appliciable.

^b^Not available.

## Discussion

### Principal Findings

According to the literature, OTC with subsequent autotransplantation provides a natural means of fertility restoration and is the only technique that can be offered to prepubertal girls [68]. For some authors, given the number of live births and ongoing pregnancies described to date, this fertility preservation method may now be considered as established [45,91]. In France, the publication of the decree dated 2008 pertaining to the rules of good clinical and biological practice for medically assisted procreation stipulates that the subsequent use of germinal tissue must remain in the field of research [100]. The publication of the June 30, 2017, decree demands that information on the state of knowledge and the results of any existing research protocols be delivered [90]. In France, the current DATOR study is one of 3 ongoing research protocols enabling the autograft of human ovarian tissue. The DATOR study is a warranty for patients as it enables wider availability of the practice of ovarian cortex autograft, notably the 2-stage grafting technique codified by Dr Pascal Piver (of the team in Limoges), by training the surgical teams in the participating centers. In addition, every case is evaluated by a multidisciplinary team.

The major concern is the reintroduction of the initial disease through malignant cells located in grafted ovarian fragments. In our project, only patients for whom the center’s multidisciplinary team and the steering committee give approval are potential candidates for autograft. This agreement takes into account the advancement of techniques evaluating residual disease at the ovarian level.

Furthermore, a study named QUALIGRAFT17 is being carried out in parallel to the DATOR protocol. QUALIGRAFT17 aims to implement complementary analyses to better assess the quality of the grafts and the risk of reintroducing malignant cells. These analyses are performed on fragments of ovarian cortex specifically reserved for the study. A first important result was achieved with the validation of multicolor flow cytometry (MFC) as a method to detect ovarian residual disease in acute lymphoblastic leukemia [96,97]. Hitherto, MFC was a customary method to identify persisting leukemic cells in blood or bone marrow [101]. Our aim is the transfer of this method to other neoplastic pathologies. Another part of this project plans to develop a functional method of qualification consisting of the identification of cells expressing markers of endothelial cells or endothelial progenitors and the assessment of the quality of the stromal cells. This functional qualification and MFC will improve the management of patients for whom risk-free ovarian autograft is possible.

### Conclusions

Data on autotransplantation after cryopreservation of ovarian tissue are increasingly encouraging, regarding both its efficacy and its safety. The results produced so far by the DATOR study are in line with such data and thus justify pursuit of this program. Our study will provide important and novel information, especially regarding early development of children born after OTC.

## Abbreviations

ABM: agence de BioMedecine (BioMedicine agency)
AMH: anti-Müllerian hormone
ART: assisted reproductive technology
DATOR: Development of Ovarian Tissue Autograft in Order to Restore Ovarian Function
FSH: follicle-stimulating hormone
LH: luteinizing hormone
MFC: multicolor flow cytometry
OTC: ovarian tissue cryopreservation
PERIDATOR: Prospective Cohort of Patients Candidates for Ovarian Tissue Autograft
